# Laccase SilA from *Streptomyces ipomoeae* CECT 3341, a key enzyme for the degradation of lignin from agricultural residues?

**DOI:** 10.1371/journal.pone.0187649

**Published:** 2017-11-07

**Authors:** Alba Blánquez, Andrew S. Ball, José Antonio González-Pérez, Nicasio T. Jiménez-Morillo, Francisco González-Vila, M. Enriqueta Arias, Manuel Hernández

**Affiliations:** 1 Departamento de Biomedicina y Biotecnología, Universidad de Alcalá, Alcalá de Henares, Madrid, Spain; 2 Centre for Environmental Sustainability and Remediation, School of Science, RMIT University, Bundoora, Victoria, Australia; 3 Departamento de Biogeoquímica, Ecología Vegetal y Microbiana, Instituto de Recursos Naturales y Agrobiología (IRNAS-CSIC), Sevilla, Spain; Universite Paris-Sud, FRANCE

## Abstract

The role of laccase SilA produced by *Streptomyces ipomoeae* CECT 3341 in lignocellulose degradation was investigated. A comparison of the properties and activities of a laccase-negative mutant strain (SilA^−^) with that of the wild-type was studied in terms of their ability to degrade lignin from grass lignocellulose. The yields of solubilized lignin (acid precipitable polymeric lignin, APPL) obtained from wheat straw by both strains in Solid State Fermentation (SSF) conditions demonstrated the importance of SilA laccase in lignin degradation with the wild-type showing 5-fold more APPL produced compared with the mutant strain (SilA^−^). Analytical pyrolysis and FT-IR (Fourier Transform Infrared Spectroscopy) confirmed that the APPL obtained from the substrate fermented by wild-type strain was dominated by lignin derived methoxyphenols whereas those from SilA^−^ and control APPLs were composed mainly of polysaccharides. This is the first report highlighting the role of this laccase in lignin degradation.

## Introduction

Laccases (benzenediol:oxygen oxidoreductase, EC 1.10.3.2) are a group of multi-copper enzymes widely distributed in nature. They have low specificity, and can oxidize a variety of phenolic and non-phenolic substrates, usually via mediators by coupling the oxidation of a substrate to the reduction of molecular oxygen to water [1]. In bacteria, the number of laccases already described is more limited than in fungi but nevertheless some of them are of great interest because of their ability to retain their function under extreme conditions which are often required by the biotechnology industry. Bacteria also exhibit higher growth rates than fungi and are more amenable to improvements in activity, selectivity and expression levels through protein engineering [2]. *In silico* biological application has provided an important impetus in discovering new robust bacterial laccases which are useful in a wide array of biotechnological applications. Since early reviews on bacterial laccases, the number of novel prokaryotic laccases that have been purified and biochemically characterized has increased significantly [2]. Among them, laccases from streptomycetes are considered an interesting alternative to fungal laccases for biotechnological purposes on the basis of their unusual structural and physico-chemical characteristics [3,4,5,6,7]. Particular interest in these enzymes is mainly due to their ability to function under different environmental conditions and also for their variation in structural protein characteristics even among different species of the same genus; for example, the thermostable small laccase (SLAC) which exhibits phenol-oxidizing activity at an unusually alkaline pH was found encoded in the genome of *Streptomyces coelicolor* [4]. Moreover, this laccase was active as a dimer when expressed in *Escherichia coli*, but as a monomer when expressed in *Streptomyces lividans* [8] and as a trimer when expressed in *Aspergillus oryzae* [9]. A disadvantage of the characterization of heterologously expressed laccases is that they bypass the biochemical characterization of a laccase in their native host making it difficult to assess the physiological role of the native laccase [2].

A number of biological functions have been proposed for bacterial laccases, including morphogenesis [3], copper homeostasis [10], pigment synthesis [11], protection against UV light and H_2_O_2_ [12] and lignin degradation [13,14,15]. Although the functions reported for bacterial laccases are numerous, in most cases the specific function of these enzymes in the native host remains unclear. To date the involvement of streptomycete laccases in lignin degradation has generally been indirectly inferred from biopulping and biobleaching applications for which the elimination of lignin from lignocellulosic substrates is mandatory. Recently, the involvement of a small laccase from *S*. *coelicolor* in lignin degradation of *Miscanthus giganteus* in submerged culture was described through the gravimetrically evaluation of klason lignin [16]. It was also reported that the main role of these microorganisms growing on such substrates under solid state fermentation (SSF) conditions was to degrade lignin and produce a high molecular weight metabolite named acid-precipitable polymeric lignin (APPL) [17,18]. The analysis of the decayed substrates by pyrolysis/gas chromatography/mass spectrometry (Py-GC/MS) demonstrated the oxidative attack of selected *Streptomyces* strains on the lignin moiety of several agricultural and woody residues [19,20]. It is important to take into account that the use of SSF systems offers advantages over submerged cultures such as the raw material do not need to be fractionated, is a low-cost method of waste management and mimics the natural environment in which these microorganisms are normally found [21].

In recent studies a thermostable laccase (SilA) produced by *Streptomyces ipomoeae* CECT 3341 showing unusual physico-chemical and structural characteristics was isolated. This enzyme shows high resistance to alkaline conditions and to high concentrations of sodium chloride [6], suggesting a robustness, which may be of commercial significance. Moreover, the enzyme contains two cupredoxine domains in its structure, as has been reported for other bacterial laccases [22]. The unusual properties of this newly isolated laccase make it worthy of further study, particularly in terms of elucidating the biological function of this laccase in the producer strain with the aim of assessing its potential for future commercial exploitation. The purpose of this work was therefore to investigate the role of the laccase SilA from *Streptomyces ipomoeae* in lignin degradation under SSF conditions, using a laccase-negative mutant strain. A comparison of the behaviour of both the wild type and the mutant strain will lead to elucidation of the role of this thermostable laccase in lignin degradation by *S*. *ipomoeae*, paving the way for assessment of the biotechnological potential of this enzyme.

## Materials and methods

### Microorganism and growth conditions

*Streptomyces ipomoeae* CECT 3341 was selected on the basis of its ability to produce the laccase SilA, already characterized in a previous study [6]. The strain was grown on Mannitol-Soy agar medium (MS) which contained mineral basal medium (MBM) [17] supplemented with mannitol, 20 g, soy flour, 20 g and agar, 20 g. For experiments, spores were harvested from MS agar plates with distilled water containing Tween 80 (0.01%). Suspensions were kept at −20°C in 20% (wt/vol) glycerol. A standard spore suspension (10^7^cfu mL^−1^) was used as initial inoculum in all assays.

To establish the time course of growth, *S*. *ipomoeae* wild-type and the laccase-negative mutant strain were grown in MBM medium supplemented with 0.6% yeast extract (w/v). Firstly, a preinoculum was obtained by inoculating 1 mL standard spore suspension (10^7^ cfu mL^-1^) in 500 mL flask containing 100 mL medium or 100 mL medium supplemented with 25 mg mL^-1^ apramycin for the mutant strain. After 48 h growth under shaking conditions (150 rpm) at 28°C, mycelia were obtained by filtration under sterile conditions. Equal portions of wild-type and laccase-negative mutant mycelia were used to inoculate 100 mL flask containing 20 mL of the media mentioned above. Flasks were incubated for 6 days under shaking conditions at 28°C and dry weight was determined by triplicate after 2, 4 and 6 days of incubation. Moreover, to check the antibiotic selective pressure, mycelium of the mutant strain was also inoculated in MBM supplemented with yeast extract without apramycin and a time course of growth was also determined for 6 days.

### Production of a laccase-free mutant

The mutagenesis of the gene encoding the *S*. *ipomoeae* laccase (*silA*) was performed by gene disruption. Firstly, an inner fragment around 600bp was amplified using the polymerase chain reaction (PCR) starting from the gene *silA* (GenBank accession DQ832180.1) using the primers Mut58-F (TGAATTCTCATCAAGATGTACGCCGAGA) located from nucleotide 151 to 170 and Mut58-R (TGAATTCCCGGTGATCTTGTTGTCGATG) located from nucleotide 771 to 792, both designed from the sequence of the gene. The PCR products (Sipo600) were flanked by EcoRI restriction sites (underlined nucleotides in the primers design) to facilitate cloning into the plasmid pOJ260 conferring resistance to apramycin. Ligation products (pOJ260-Sipo600) were transformed into an electrocompetent strain from *E*. *coli* ET12567 (pUB307) by electroporation. An electroporated colony was then conjugated with spores of *S*. *ipomoeae*, with the subsequent selection for apramycin resistant spores (SilA^−^) [23].

### Molecular and biochemical verification of *S*. *ipomoeae* mutant (SilA^−^)

The disruption of the *silA* gene was confirmed by PCR. To confirm the absence of the laccase gene in SilA^−^ mutants a PCR of the genomic DNA from both *S*. *ipomoeae* wild-type and mutant strains was carried out using the primers SilA-F (TAGGTATGACCGGGATGGAA) and SilA-R (GAGGTGCAGGGAGAAGACC) flanking the gene *silA*. Subsequently, the amplification product was sequenced by Sanger sequencing. The absence of laccase activity in the mutant strain was confirmed in liquid and solid MBM culture medium supplemented with ABTS (2,2'-azino-bis-(3-ethylbenzothiazoline-6-sulphonic acid) as enzyme substrate. In liquid medium, a standardized spore suspension from both wild and mutant strains was used to inoculate 500 mL flasks containing 100 mL of MBS. The flasks were incubated at 28°C for seven days. Daily, a 1 mL sample was taken from each flask in order to check enzyme activity. Laccase activity was determined at room temperature by measuring the oxidation of 5 mM ABTS in 50 mM acetate buffer (pH 4.5). The increase in absorbance at 436 nm for ABTS was monitored using a Hitachi 2001 spectrophotometer and a molar extinction coefficient of 29300 M^-1^ cm^-1^ for oxidized ABTS [24]. For the detection of laccase activity in solid medium, the same spore suspension was placed in spots on MBS culture agar supplemented with 1 g L^-1^ of ABTS. After 4 days of incubation the presence of a green halo around the colonies was indicative of the production of the enzyme.

### Complementation of laccase-negative mutant (SilA^−^)

The *S*. *ipomoeae* SilA- strain was complemented using the *silA* laccase gene cloned into the plasmid pEM4T, a bifunctional *E*. *coli*-*Streptomyces* plasmid, under the control of the ermE* promoter. The gene *silA* (1-kb) was amplified by PCR using the primers CM58-F (AAAGGATCCGAGCGTGGGGAGTT, BamHI underlined) and CM58-R (AAAGAATTCCTCGCCACCGTCCG, EcoRI site underlined). The 1-kb fragments obtained from the PCR, Sipo1000, were cloned into the plasmid pEM4T conferring resistance to thiostrepton. The construction pEM4T-Sipo1000 was purified and used for the electroporation of an electrocompetent strain of *E*. *coli* ET12567 (pUB307). An electroporated colony was then conjugated with spores of *S*. *ipomoeae* SilA^−^ with the subsequent selection with apramycin (25 μg mL^-1^) and thiostrepton- (25 μg mL^-1^) resistant spores. A copy of the laccase gene was also introduced following the same methodology into the wild-type strain of *S*. *ipomoeae* to verify if an over-expression of SilA laccase occurs (super wild-type strain).

Both complemented mutant and super wild-type strains were cultivated in 20 mL MBM medium supplemented with 1% mannan, 100 μM of copper sulphate and 0.2% asparagine. Apramycin and thiostrepton were added at 25 μg mL^-1^ and cultures were maintained at 28°C and 150 rpm for 4 days. Laccase activity was daily measured. All cultures were carried out in triplicate.

### Solid-state fermentation (SSF) of wheat straw

In order to determine the role of SilA laccase in lignin degradation a solid-state fermentation (SSF) process using wheat straw as substrate was performed. Wheat straw (*Triticum aestivum* var. maestro) was ground in a Janke and Kunkel A-10 mill to pass through a 40-mesh screen and air-dried for 24 h at 50°C. To facilitate the colonization of the substrate unplugged 2 L flasks containing 10 g wheat straw were steamed for 1 h. The flasks were plugged with cotton stoppers and autoclaved for 20 min at 120°C. Pre-inocula of wild and mutant strains were obtained by growing standardized spore suspensions (10^7^cfu mL^-1^) in MBM medium pH 7 supplemented with 0.6% (w/v) yeast extract and 1 M sucrose. For growing the mutant strain 25 μg mL^-1^ apramycin was also added. The cultures were incubated for 36 h at 28°C with shaking (200 rpm) and the mycelia harvested by centrifugation (10,000 *g*, 15 min). To facilitate the colonization of wheat straw, inocula were prepared by resuspending the mycelia in 50 mL MBM medium supplemented with 3.5% (w/v) NaCl and 0.1% (w/v) yeast extract. In the case of SilA^−^ strain an apramycin solution (25 μg mL^-1^) was also added. The cultures were then incubated statically for 7 d and uninoculated controls were also incubated at the same conditions.

Active biomass of both wild-type and mutant strains was estimated by the CO_2_ released during the growth of microorganisms on wheat straw using NaOH traps. NaOH (20 mL 0,1 M in sterile containers) were introduced into the flasks where the fermentation of the substrate was carried out. CO_2_ production was determined after 6 days of incubation by precipitating the dissolved carbonates as barium carbonate and further titration with HCl [25].

### APPL extraction and lignin:carbohydrate ratio determination

After 7 d growth in SSF, the acid-precipitable polymeric lignin (APPL) from solid-substrate fermentation cultures was extracted with distilled water. Samples were steamed at 100°C for 1 h, filtered through Whatman no. 54 filter paper and washed with distilled water at 60°C. Supernatants were acidified with HC1 (12 M) to pH 1–2 and the APPL harvested by centrifugation (12000 g, 10 min). Finally, the APPL was washed twice with distilled water (pH 5) and freeze-dried. The yield of APPL was expressed as mg of freeze-dried APPL per g wheat straw.

To determine the lignin-carbohydrate ratio of the APPLs obtained from uninoculated wheat straw and from those obtained following the growth of the wild-type and mutant strains, 10 mg of each APPL were resuspended in 100 mL 0.1M NaOH. Ethanol (100 mL) was added to precipitate the carbohydrates [26] and the mixture maintained at 4°C for 12 h. Samples were centrifuged at 9,000 rpm for 20 min and the residue lyophilized and weighted. Supernatants were then acidified to precipitate the lignin as described in the above section.

### Structural characterization of APPL

#### Pyrolysis-gas chromatography/mass spectrometry (Py-GC/MS)

The APPL (1 mg) extracted from control and fermented wheat straw by wild type and mutant strains was analysed by analytical pyrolysis (Py-GC/MS) in a microfurnace pyrolyzer (Model 2020, Frontier Laboratories) connected to a GC-MS system (Agilent 6890) fitted with a fused silica capillary column HP 5MS (30 m x 250 μm x 0.25 μm inner diameter) as described in [27]. Lignin and polysaccharide pyrolysis products were identified on the basis of major ions detected in the average mass spectra in the region where the main biogenic compounds are known to elute as previously established [27]; *i*.*e*. the initial phase (2–5 min) is dominated by polysaccharide-derived compounds and the latter phase (5–14 min) by lignin-derived compounds including G, S, and H derived units.

#### Fourier Transform Infrared (FT-IR) spectroscopy

The infrared spectra (wavenumber range of 4000–600 cm^−1^) were obtained from KBr pellets containing approximately 1% (w/w) of APPL powdered sample prepared in a cylindrical piston under high pressure and vacuum. The spectrophotometer used was a JASCO model 4100. The spectra were acquired by averaging 60 interferograms at 2 cm^−1^ resolution for each recorded spectrum. Spectral data were background corrected against a pure KBr pellet reference spectrum prior to every measurement. Initial data manipulation was performed using JASCO spectra manager. In order to prevent subjective baseline values and to enhance the resolution, the original spectra were modified by subtracting a positive multiple of its 2^nd^ order derivative [28]. The assignment of signals was done in accordance with previous studies with lignocellulosic material [29,30,31,32,33].

### Statistical analysis

Statistical significance of the differences obtained between the growth (*i*.*e*., dry weight) of wild-type and mutant strains in SSF conditions were analysed using a GLM univariate procedure. Time and culture medium were included as factors. In addition, the differences in the APPL amount obtained and in the CO_2_ produced between the wild-type and mutant strains were statistically analysed using an unpaired *t*-test. All experimental data were analyzed using Statistica v8.0 software.

## Results

### Production of the laccase-negative mutant

**Fig 1:**
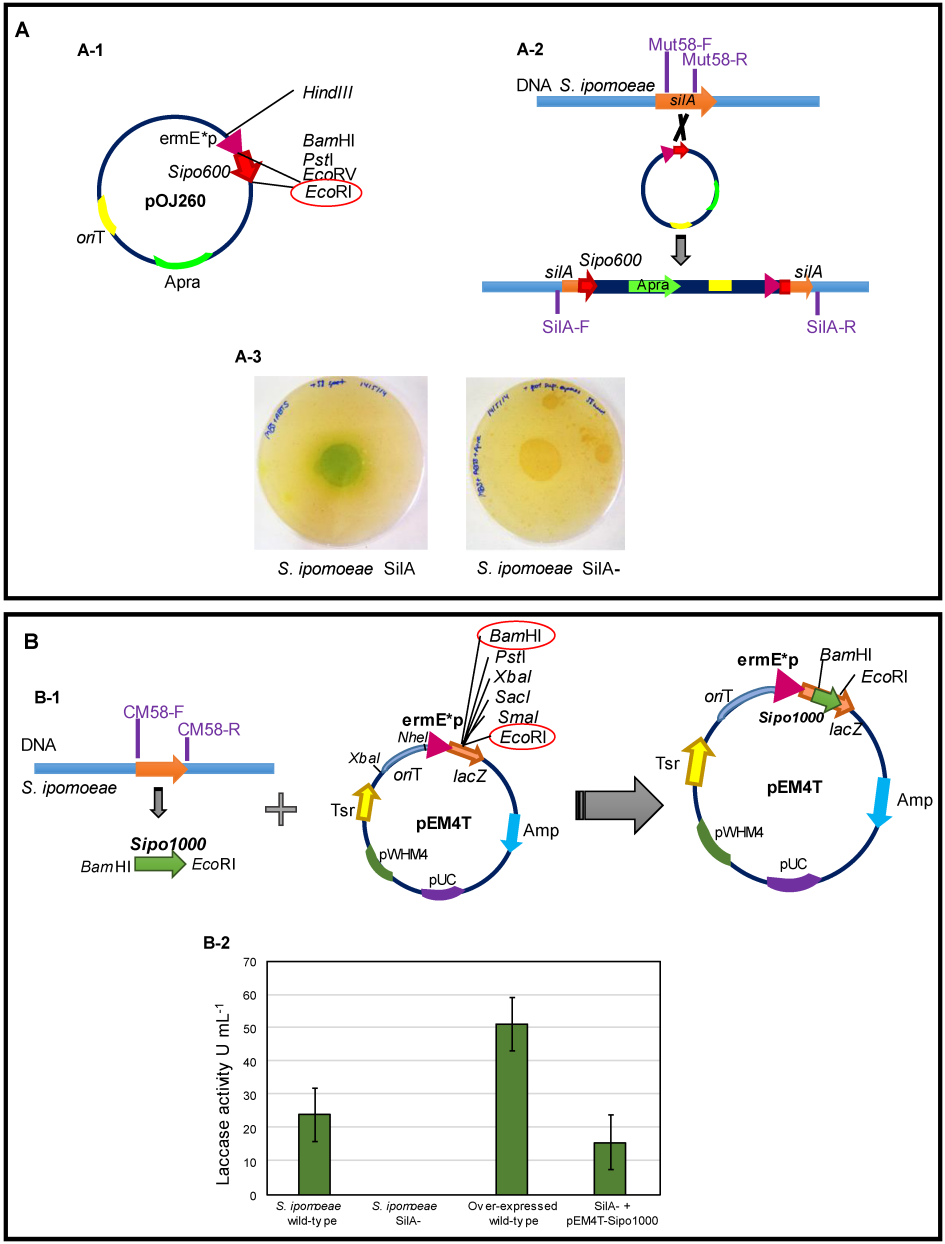
**A**; pOJ260-Sipo600 construction (A-1), scheme showing the insertion of the pOJ260-Sipo600 construction in the *S*. *ipomoeae* genome by simple crossover (A-2) and photograph showing the production of laccase activity in ABTS-solid media by the wild-type strain (SilA) and the absence of laccase activity in the mutant strain SilA^−^ (A-3). **B**; Production by PCR of the Sipo1000 fragment and insertion into the pEM4T plasmid between BamHI and EcoRI restriction sites. (B-1), laccase activity determined from *S*. *ipomoeae* wild-type, mutant SilA^−^, recovered mutant (SilA^−^ + pEM4T-sipo1000) and over-expressed wild-type (B-2).

**Fig 2:**
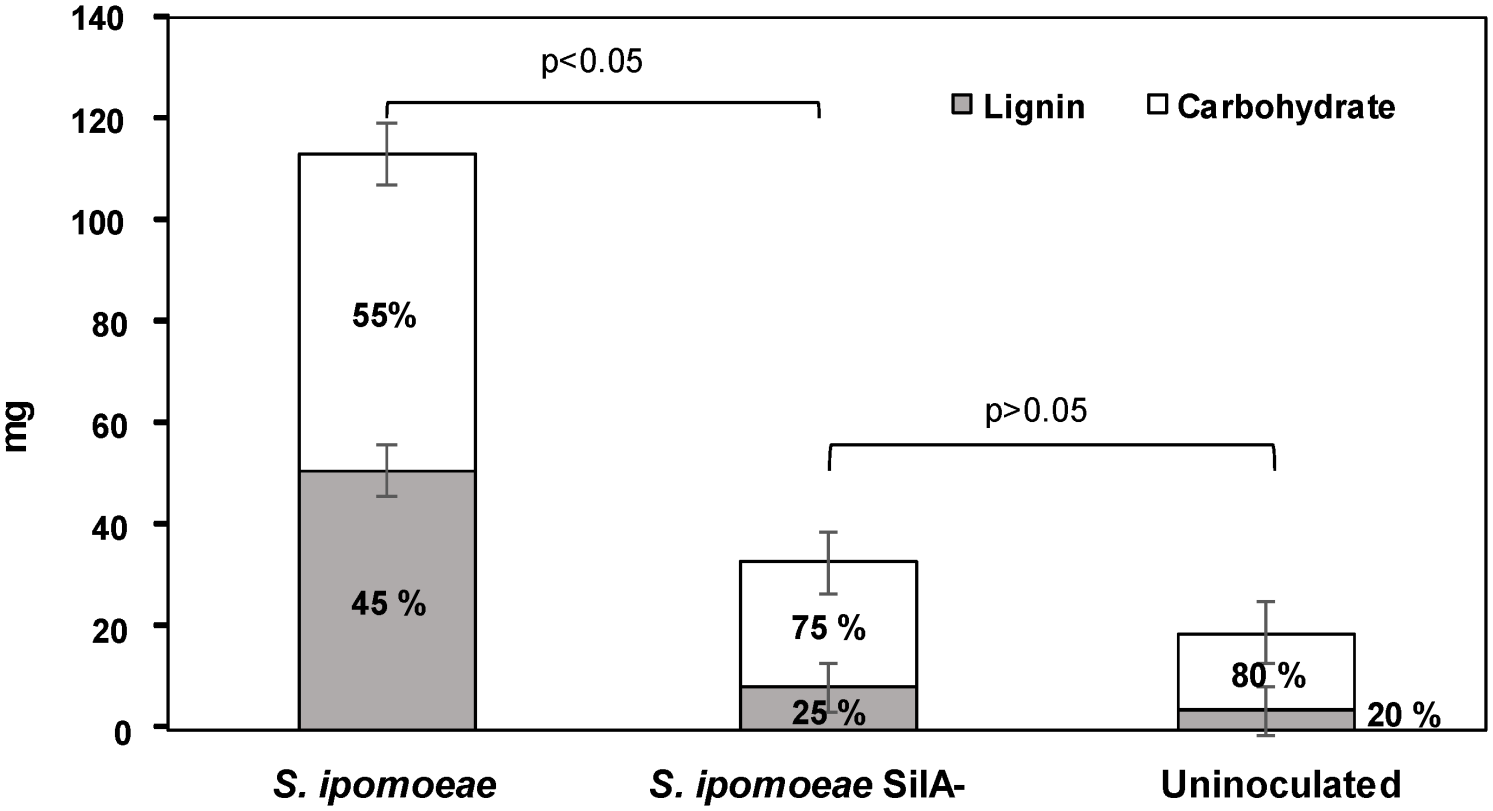
Total production of APPL and lignin:carbohydrate ratio obtained from wheat straw fermented with *S*. *ipomoeae* wild-type and mutant strains and from an un-inoculated wheat straw.

To obtain a laccase-negative mutant of *S*. *ipomoeae* gene disruption was carried out by insertion of an apramycin-resistant cassette. For this, the amplification of a 600 bp fragment from the *silA* gene and its later insertion in the pOJ260 vector was performed. This fragment is long enough to recombine only with the same sequence in the gene *silA*, avoiding non-specific recombination with other genes of the genome. The schematic representation of the construction is showed in Fig 1A-1. The insertion of pOJ260-Sipo600 from *E*. *coli* into the *S*. *ipomoeae* genome by simple crossover is represented in Fig 1A-2. To avoid possible polar effects caused by gene disruption, the activity of promoter ermE*p from the plasmid allows transcription of genes downstream of the integration site.

**Fig 3:**
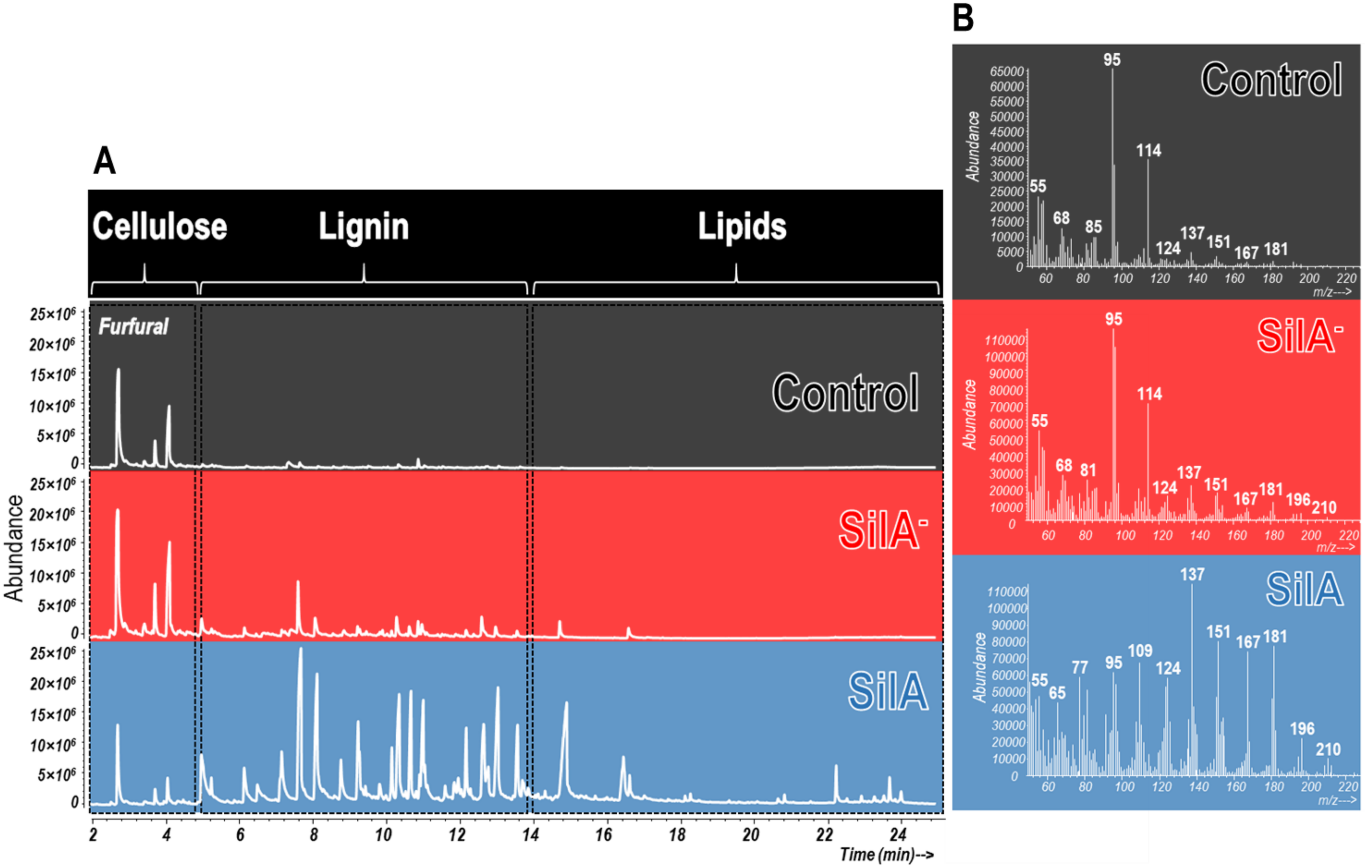
(A) Py-GC/MS chromatograms of APPL obtained from control and wheat straw transformed in SSF conditions by wild-type (SilA) and laccase-negative mutant (SilA^−^) strains; the main domains where the different precursors are eluted are indicated. (B) Average Py-EI-MS spectra corresponding to the domains where pyrolysis polysaccharides (cellulose) and lignin derived compounds elute (min 2 to min 14 of the chromatograms).

The correct interruption of the SilA laccase gene was checked by molecular and biochemical screening. For the molecular screening, the DNA of both mutant and wild-type strains were amplified using SilA-F and SilA-R primers which flank the gene *silA*. The results showed that amplification in the SilA^−^ strain was around 5 kb and in the wild-type strain 1 kb, corroborating the presence of the pOJ260 (3.5 kb) plasmid in the mutant strain. This result was also confirmed by biochemical screening in solid media containing ABTS as substrate for the laccase. The results showed the presence of a green halo around the colonies of the wild strain as a result of the oxidation of the ABTS by the SilA laccase but not in the mutant strain (Fig 1A-3). The laccase activity assays carried out in liquid cultures allowed detection of up to 24 mU mL^-1^ activity in the supernatant of the wild-type strain after 4 days of incubation. However, no activity was detected in the supernatant of the laccase-negative mutant over the time course of incubation.

The production of the laccase activity in the mutant strain was restored by complementation with the original *silA* gene inserted into the pEM4T plasmid (Fig 1B-1). We observed the recovery of the laccase function in the complemented mutant strain by the detection of 15.7 ± 1.5 mU mL^-1^ activity in liquid cultures compared to that detected in the wild-type culture (24 ± 1.9 mU mL^-1^). Moreover, the introduction of an additional copy of the *silA* gene into the wild-type strain yielded an overexpressed strain producing double laccase activity (51 ± 4.08 mU mL^-1^) than the wild-type strain (Fig 1B-2).

### Time course of growth of the mutant SilA^−^ in complex medium

In order to assess the viability of the mutant strain, a time course of growth of this strain was carried out in a complex medium (MBM + YE) and the results compared with those obtained from the wild-type. Moreover, to determine whether the presence of apramycin is a limiting growth factor the time course of growth was also performed in the presence and absence of this antibiotic. The maximum growth was obtained in all cases after 4 days of incubation reaching values of 29.3, 20.7 and 22.2 mg dry weight for the wild-type strain, mutant strain plus apramycin and mutant strain without apramycin, respectively. Although the mutant strain in the presence of the antibiotic showed a lower dry weight, the differences found with wild-type and the mutant strains without apramycin were not significant (p>0.05).

### Involvement of SilA in lignin degradation

Solid state fermentation (SSF) of wheat straw by wild-type and mutant strains revealed differences in the growth of both strains. While the wild-type strain exhibited normal growth and sporulation, both processes were less defined in the SilA^−^ mutant strain. The amount of water extracted APPL from the substrates after 7 days of incubation was 113 mg g^-1^ from wild type strain culture, significantly greater than the 33 mg g^-1^ from SilA^−^ culture (p<0.05). From the uninoculated control straw the yield extraction was just 19 mg g^-1^ a value which was not significantly different to the value obtained from the mutant strain (p>0.05). Moreover, the lignin carbohydrate ratio in these APPLs also differs between the wild-type and the mutant strains. Thus, this ratio for the mutant strain was 25:75 (Fig 2), quite similar to that obtained from the APPL extracted from uninoculated wheat straw (20:80). In contrast, the APPL obtained from the wild-type strain showed a much higher proportion of lignin (45:55 lignin:carbohydrate ratio).

To assess the impact of the absence of the laccase on the ability of the strain to produce APPL and utilise lignocellulosic material as the main energy source, the growth of the wild type and SilA^−^ strains were compared after 6 days through assessment of respiratory activity. Total CO_2_ production determined in wild-type cultures was 123.8 ± 4.5 mg CO_2_ per gram wheat straw, significantly greater than the 74.9 ± 2.7 mg CO_2_ per gram wheat straw produced by the laccase negative strain (p>0.05).

The pyrograms corresponding to APPL obtained from the control and fermented wheat straw by the wild-type (SilA) and laccase-negative mutant (SilA^−^) strains are shown in Fig 3A. The chromatograms can be divided in three main areas where the main pyrolysis products from well-known biogenic precursors elute: polysaccharides (2.0–4.4. min), lignin (4.4–14 min) and lipids (14 to 25 min). In the chromatogram from the wild-type SilA strain, pyrolysis compounds derived from lignin are also clearly distinguishable. In addition, some fatty acids (mainly C14 and C16) and sterols are also detected.

The Py-EI-MS average spectra (min. 2 to min. 14), showing the dominant compounds in each sample are shown in Fig 2B. The control APPL and that extracted from wheat straw fermented by SilA^−^ are clearly dominated by polysaccharide thermal oxidation products *i*.*e*. furfural (m/z 95, 96) and pentoses (di-anhydropentoses) (m/z 85, 114). In addition, signals from monomeric pyrolysis products of lignin with guaiacyl (m/z 124, 137, 151) and syringyl (m/z 167, 181) structures could also be detected, but only at very low concentrations when compared to those corresponding to APPL from wheat straw fermented by the SilA laccase producing strain. The latter was conspicuously dominated by signals from lignin subunits consisting also of guaiacyl (m/z 124, 135, 137, 138, 151), syringyl (m/z 154, 167, 181, 194, 208) and also *p*-hydroxyphenyl structures (m/z 107).

**Fig 4:**
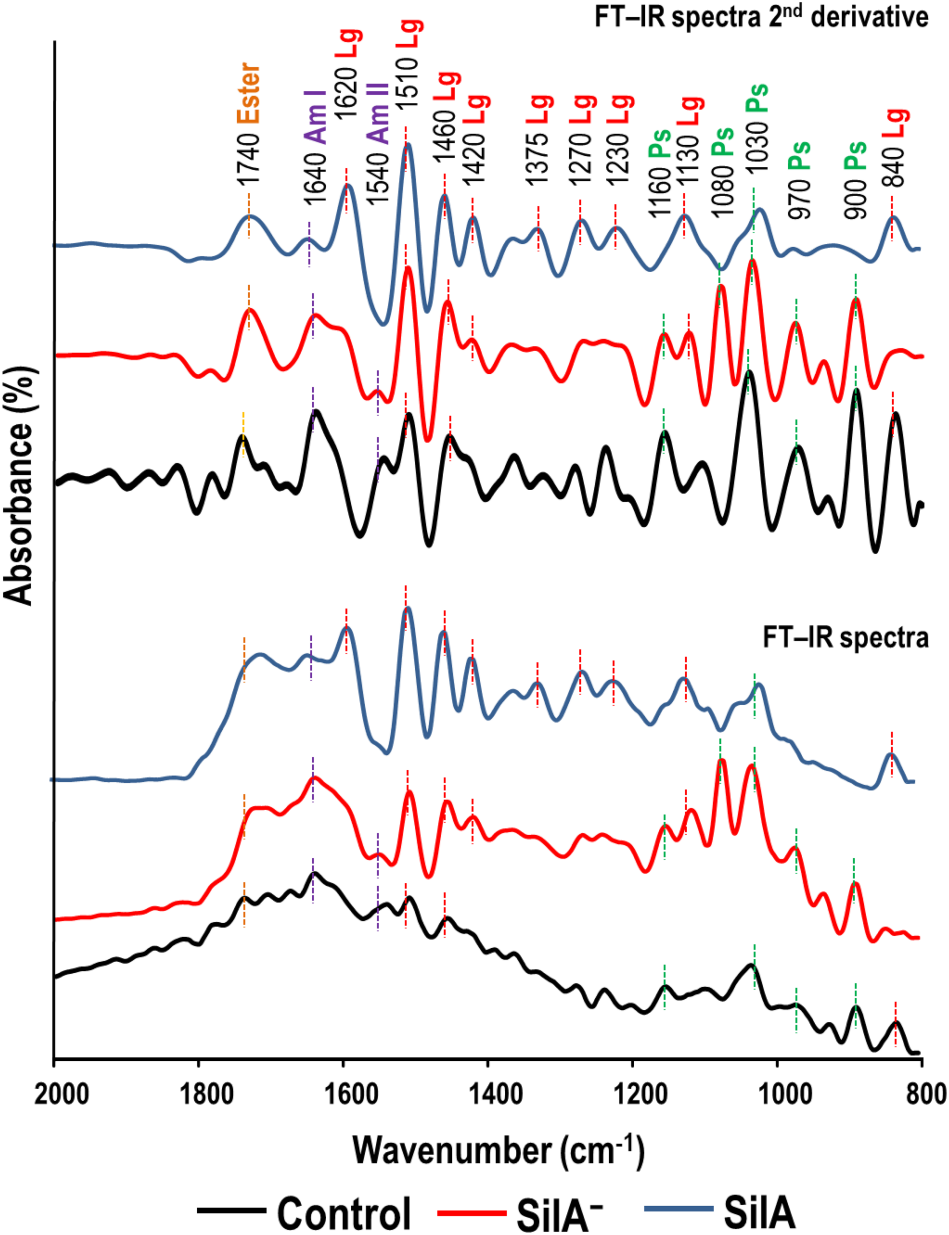
FT-IR spectra of APPL obtained from control wheat straw and transformed in SSF conditions by wild-type (SilA) and laccase-negative mutant (SilA^−^) strains. Labels on the peaks corresponds to wavenumber of peaks in cm^-1^ followed by assignation as ester; amide I (Am I); amide II (Am II); lignin and polyphenols (Lg); polysaccharides (Ps).

FT-IR spectroscopy was applied to the same samples analyzed by Py-GC/MS to obtain parallel qualitative and quantitative information on the differences between the APPL obtained from wheat straw treated with the wild-type (SilA) and the laccase-negative mutant (SilA^−^) strains. APPL IR spectroscopy revealed details on the role of laccase in lignin solubilization. Fig 4 shows the most informative part of FT-IR spectra (800–2000 cm^-1^) obtained from APPL extracts from the control wheat straw and SSF transformed with the wild-type (SilA) and the laccase-negative mutant (SilA^−^) strains. In spite of the heterogeneity of the APPL extracts, and except for the control, the spectral patterns have absorption bands compatible with lignocellulosic material. The intensity of the different FT-IR bands is indicative of the relative concentration of the major lignocellulose constituents as well as of protein from the *Streptomyces* strains.

With respect to the cellulose (polysaccharide) domain, signals at ca. 1160, 1080, 1030, 970 and 900 cm^-1^ were more prominent in the control APPL and that from the SilA^−^ strain. Specifically, the signals at ca. 1080–1030 are usually assigned as C-O vibrations in cellulose pyranoside units and that centred at ca. 900 cm^-1^ with C-H deformations in cellulose. The assignation of signals at ca. 1030 is not clear and apart to the cellulose domain is also frequently assigned to silicate (Si-O-Si) or to methoxyl groups in guaiacol rings ([30, 32] and [34]).

In contrast, the signals from the lignin (polyphenolic) domain are clearly prominent in the APPL from the wild-type strain (SilA). In particular, the presence of lignin is shown by a well-defined pattern with a characteristic band at ca. 1510 cm^-1^ and additional peaks at 1460, 1420, 1375, 1270 and 1230 cm^–1^ e corresponding to different substitutions in the aromatic methoxyphenolic lignin units [29]. Other diagnostic spectral bands at ca. 1650 cm^-1^ (amides I) and ca. 1540 cm^-1^ (amides II) indicate the presence of residual protein from microbial biomass. The band at ca. 1740 cm^-1^ can be attributed to C = O stretching in association with carboxylic groups or esters in hemicellulose.

All the differences in the composition of the APPLs described above become more evident after the analysis of the second-derivative FTIR spectra. Distinct differences can be seen between the various bands associated with lignin and carbohydrates; the intensity of the lignin peaks at 1510, 1620 and 1460 cm^-1^ in the APPL obtained from the wild-type strain confirm the higher lignin content of these samples compared with that of the laccase-negative mutant strain, again confirming a significantly reduced lignin solubilizing activity compared with the wild-type strain.

## Discussion

It seems clear that bacterial laccases have a variety of functions, suggesting that these enzymes are functionally diverse and worthy of further research. In particular, the role of laccases in lignin degradation by streptomycetes remains unclear despite the importance of lignin degradation both in the environment and commercially in biofuel production. Nevertheless, in some studies the role of these enzymes in physiological processes has been reported. Laccases from *Streptomyces* such as the ScyA laccase from *Streptomyces cyaneus* [24] and the SLAC laccase from *S*. *coelicolor* [4,16] have been reported to be involved in lignin degradation, although the chemical structure of the solubilized lignin and the role of laccases in modifying lignin from natural substrates has yet to be confirmed. Here, the approach to the study of the biological role of the SilA laccase from *S*. *ipomoeae* was to generate a laccase-negative mutant followed by subsequent comparison of the behaviour of this mutant with the wild-type strain in terms of their relative lignin degrading capability under SSF conditions.

Previous studies suggested the importance of ligninolytic enzymes including laccases from streptomycetes in the degradation of lignin from lignocellulosic residues [17]. The extracellular laccase produced by *S*. *cyaneus* has been previously shown to degrade lignocellulosic residues under solid state fermentation (SSF) and also to delignify kraft pulps, but only in the presence of ABTS as a mediator [24]. Further studies demonstrated the ability of several *Streptomyces* strains to oxidise lignin from pine wood under SSF conditions [20]. More recently, a new thermostable, pH-versatile and salt-resistant laccase produced by *S*. *ipomoeae* was shown to detoxify a textile dye azo- and quinolone-based antimicrobials, both in the presence of acetosyringone as a redox mediator [6,35] and to delignify eucalyptus kraft pulps also in the presence of the same mediator [36]. Based on these promising results, the possible involvement of this laccase in lignin degradation from of a natural residue, wheat straw, was explored.

This study significantly extends previous work in that it focuses on confirming the role of laccases in the natural lignin degradation process. In order to obtain more accurate information about the role of the laccase SilA on grass lignin degradation the lignin solubilized product (APPL) from uninoculated control and fermented wheat straw by the wild type (SilA, containing laccase) and mutant (SilA^−^, no laccase activity) strains was analyzed by using both degradative (Py/GC-MS) and non-degradative (FT-IR spectroscopy) techniques. Quantitative estimation of APPL showed that the lignin solubilization yield of the wild type strain were 5 and 12-fold higher than that determined for the mutant strain and the control respectively. These results differ from those reported by Majumdar et al. (2014) who found that the difference of APPL yields between *S*. *coelicolor* A3(2) wild-type and a laccase negative mutant strains was around 1.6-fold. Our findings show up the highest effectiveness of *Streptomyces* laccases in SSF conditions. In fact, it was recently described the advantage to use SSF systems over submerged cultures due to the higher enzyme productivity [37].

Moreover, the lignin:carbohydrate ratio obtained from the extracted APPL showed differences between both strains. The wild-type strain not only solubilized more APPL than the mutant strain, but almost half of the APPL obtained corresponds to lignin, compared with just 25% lignin in the APPL from the mutant strain. This result was confirmed by Py/GC-MS and FTIR analyses. The fact that laccase negative mutant strain degrades more lignin than the control could be attributed to the production of other lignocellulolytic enzymes, such as xylanases, ferulic acid esterases, peroxidases, etc already described in other *Streptomyces* strains [19]; however, the action of low molecular weight radicals (mediators) could be discarded because of the absence of laccase activity in the mutant strain. These results confirm for the first time a direct role for SilA laccase in lignin degradation from lignocellulose under SSF conditions. It could be considered that reduced APPL production was merely a result of reduced growth; however, whilst studies did show that the respiration rate assessed during growth on wheat straw was reduced by 40% in the mutant strain, compared to the wild-type, APPL production was only 20% of APPL compared with the wild-type. The ratio of APPL production per unit CO_2_ was 0.96 mg APPL per mg CO_2_ for the wild-type while this ratio was just 0.45 for the mutant strain. These results reinforce that mutant strain is less efficient degrading lignin than the wild-type. All these data point out the important role of SilA laccase in lignin degradation.

The pyrograms and FT-IR spectra showed significant differences among the APPLs analyzed. In the APPL obtained from the substrate fermented by the SilA strain lignin-derived compounds are the main components, while for the SilA^−^ strain, as well as for the control, APPL compounds derived from polysaccharides were dominant. The results obtained from both analytical techniques confirm the high lignin solubilizing activity of the laccase SilA and demonstrates the important role of this enzyme in lignin degradation of wheat straw.

The involvement of a streptomycete laccase in lignin degradation was also recently reported using a laccase-less mutant of *S*. *coelicolor* A3(2) and *Miscanthus giganteus* as lignocellulosic substrate in submerged culture [16]; however, importantly, comparative analysis of the APPLs was based on the determination of the klason lignin content. This does not allow qualitative differences between the wild-type and mutant strains to be inferred. However, in the current study analytical pyrolysis and FT-IR did enable specific qualitative and quantitative differences to be established.

In summary, comparison of the behaviour of a laccase-negative mutant strain with the wild type *S*. *ipomoeae* provides, for the first time direct evidence that the presence of the thermostable, pH-versatile and alkali-resistant laccase SilA results in significant lignin degradation of a native lignocellulosic substrate in a short incubation period (seven days) as seen in a simple water soluble lignin extractions obtained directly from the solid growth substrates. The role played by this laccase confirms the potential of this enzyme for use in industries for which the total or partial elimination of lignin is mandatory, such as the pulp and paper industry and biorefineries.
